# Optical Bloch oscillation and Zener tunneling in the fractional Schrödinger equation

**DOI:** 10.1038/s41598-017-17995-7

**Published:** 2017-12-19

**Authors:** Yiqi Zhang, Rong Wang, Hua Zhong, Jingwen Zhang, Milivoj R. Belić, Yanpeng Zhang

**Affiliations:** 1 Key Laboratory for Physical Electronics and Devices of the Ministry of Education & Shaanxi Key Lab of Information Photonic Technique, Xi’an Jiaotong University, Xi’an, 710049 China; 2 Department of Applied Physics, School of Science, Xi’an Jiaotong University, Xi’an, 710049 China; 3 Science Program, Texas A&M University at Qatar, P.O. Box 23874 Doha, Qatar

## Abstract

We demonstrate optical Bloch oscillation (OBO) and optical Zener tunneling (OZT) in the fractional Schrödinger equation (FSE) with periodic and linear potentials, numerically and theoretically. We investigate in parallel the regular Schrödinger equation and the FSE, by adjusting the Lévy index, and expound the differences between the two. We find that the spreading of the OBO decreases in the fractional case, due to the diminishing band width. Increasing the transverse force, due to the linear potential, leads to the appearance of OZT, but this process is suppressed in the FSE. Our results indicate that the adjustment of the Lévy index can effectively control the emergence of OBO and OZT, which can inspire new ideas in the design of optical switches and interconnects.

## Introduction

In 1929, F. Bloch predicted that electrons in a periodic potential excited with a dc external electric field will exhibit an oscillating rather than a straight accelerating behavior^1^. This phenomenon is the now well-known Bloch oscillation, named after him. It remained a fundamental problem in quantum mechanics and a classical prediction that was not verified for more than 60 years. An experimental observation was achieved only in 1992, by Feldmann *et al*.^2^. In that pioneering research, the authors experimentally reported Bloch oscillation in a semiconductor superlattice by means of a transient degenerate four-wave mixing–a kind of optical process. Thus, Bloch oscillations were first observed in optics, in a photonic device; direct observation of Bloch oscillations of electrons in quantum solid state is still an open problem. For optical observation, a few ingredients were necessary: First, a periodic potential with an external dc field that can be prepared by elaborately designing a photorefractive index change or the shape of waveguide arrays in a photonic crystal. Second, a paraxial wave equation describing the propagation of beams in this system that is formally equivalent to the Schrödinger equation describing the system of oscillating electrons. As a result, Bloch oscillations were quite intensely investigated in optics in the last few decades, and this interdisciplinary research opened a new avenue for checking quantum theorems that are difficult to prove in quantum mechanics but are easy to prove in optics. This approach constitutes now the field of quantum-optical analogies^3^.

The convenience of quantum-optical analogies is that they map the temporal evolution of wave functions in quantum phenomena onto the spatial propagation of optical fields in photonic devices. As such, they display great applicative potential for making interesting new photonic devices such as beam combiners, splitters and interferometers. Concerning the optical Bloch oscillation (OBO), the related literature is available in a large supply, and most of the important results are presented in the few recent review papers^3–6^. Until now, OBO has been reported in but is not limited to cold atoms^7–11^, optical waveguides^12–15^, photonic lattices^16–21^, integrated photonic circuits^22,23^, and non-Hermitian systems^24,25^. However, to the best of our knowledge, Bloch oscillations in the fractional Schrödinger equation were not investigated before. This task is undertaken in this paper.

The fractional Schrödinger equation (FSE) is the fundamental equation of the fractional quantum mechanics^26–28^. Compared to the standard Schrödinger equation, it features the fractional Laplacian operator instead of the regular one. This substitution brings a profound change in the behavior of the wave function. Until now, most of the research on FSE was focused on mathematical issues and the steady behavior of wave packets in simple potentials. However, even the relativistic massless harmonic oscillator^29–31^ associated with FSE turned out to be not so simple. The difficulty stems from the fact that the fractional Laplacian operator, which sits in the FSE instead of the ordinary Laplacian, is inherently a nonlocal mathematical operator.

The FSE was introduced in optics in 2015, by S. Longhi^32^. In that work, an aspherical optical cavity was designed to realize FSE, and the dual Airy function^31,32^ was demonstrated to be the eigenmode of the massless harmonic oscillator. After that work, FSE with a harmonic potential was reported^33^, in which the beam propagated according to a zig-zag trajectory and formed a filament-like structure during propagation. FSE with a periodic potential was also investigated^34^, and conical diffraction was demonstrated, due to the band structure becoming linear at certain locations. Since the fractional Laplacian causes non-parabolic dispersion, which suggests the possibility of directly modulating the dispersion of a physical system, it is not easy to find real physical systems described by the FSE. To overcome this difficulty, a potential link between the FSE and the beam propagation in honeycomb lattice was established, based on the Dirac-Weyl equation^35^. This represented one of the first attempts to identify a real physical system that can be described by the FSE. Also, a proposal for realization of the free FSE (without a potential) is made by utilizing the Fourier transform method^36^. Nowadays, investigations of the FSE are numerous, and interesting phenomena based on the FSE are reported^37,38^. We should note that the mentioned literature mainly concerns the linear FSE. But recently, the nonlinear FSE is also becoming intensely investigated, with a wealth of nonlinear effects reported^39–42^.

Based on the variety of models and exciting progress on OBO and FSE, in this paper we connect these two fundamental phenomena, and demonstrate the emergence of OBO in FSE. We also show how the linear potential brings the appearance of OZT in the same system. To the best of our knowledge, these topics have never been discussed before. As a reference, we first discuss the OBO and OZT in the regular Schrödinger equation, and then by adjusting the Lévy index *α*, switch to the FSE. Since the fractional Laplacian operator brings novel features that are absent from the regular Laplacian operator, the OBO and OZT in the FSE deserve adequate attention and promise to bring novel interesting phenomena. We believe that our research will not only enrich the OBO and OZT family of phenomena, but also inspire new ideas in the research of FSE.

## Results

### Optical Bloch oscillation

**Figure 1:**
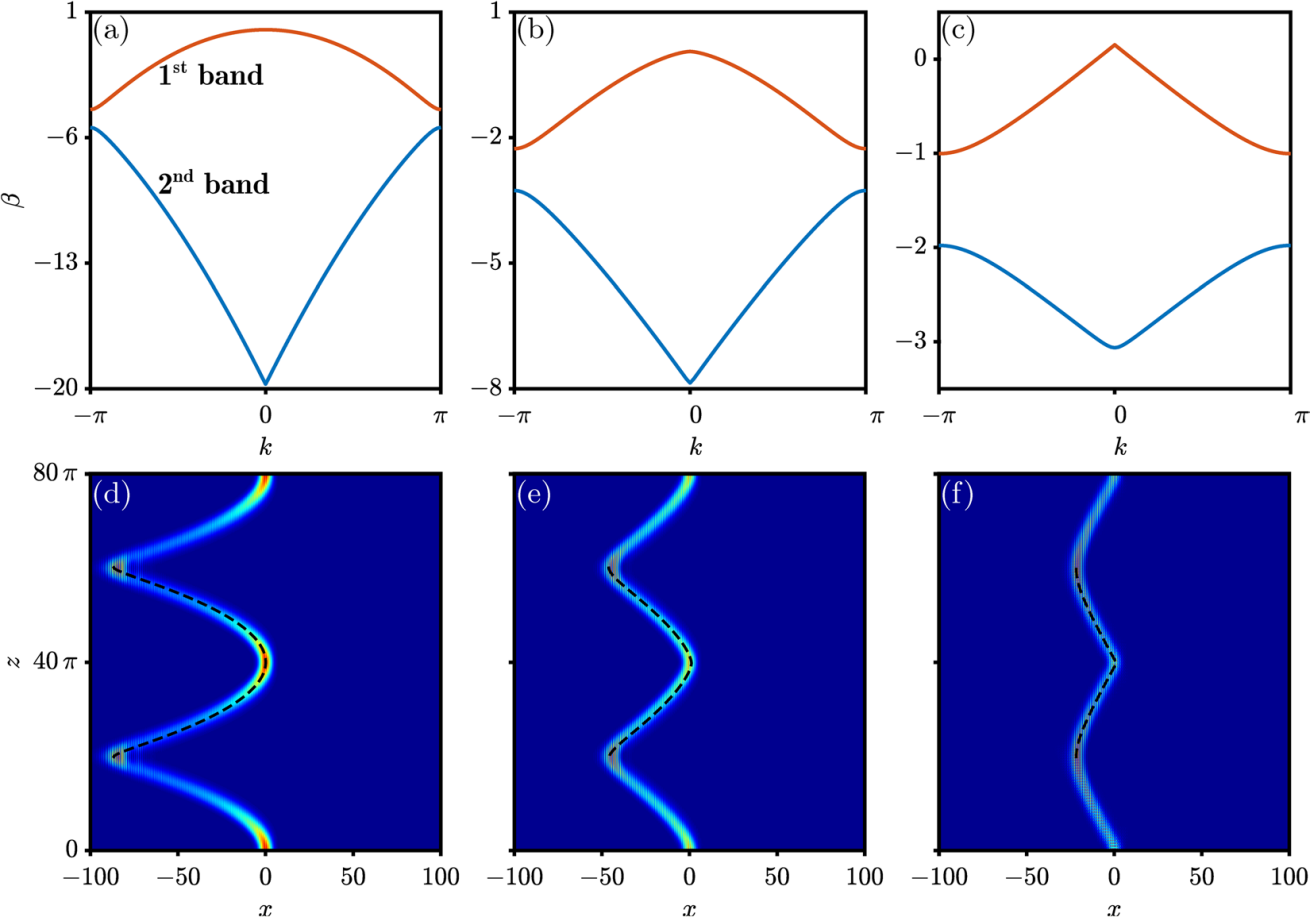
Band structure and optical Bloch oscillation. (**a**)–(**c**) Band structures corresponding to *α* = 2, 1.5 and 1, respectively. (**d**)–(**f**) Optical Bloch oscillations corresponding to (**a**)–(**c**). The dashed curves in (**d**)–(**f**) indicate the first (orange) band in (**a**)–(**c**). Other parameters: *a* = 0.05, *d*_0_ = 1, and ${\mathscr{D}}=40\pi$.

**Figure 2:**
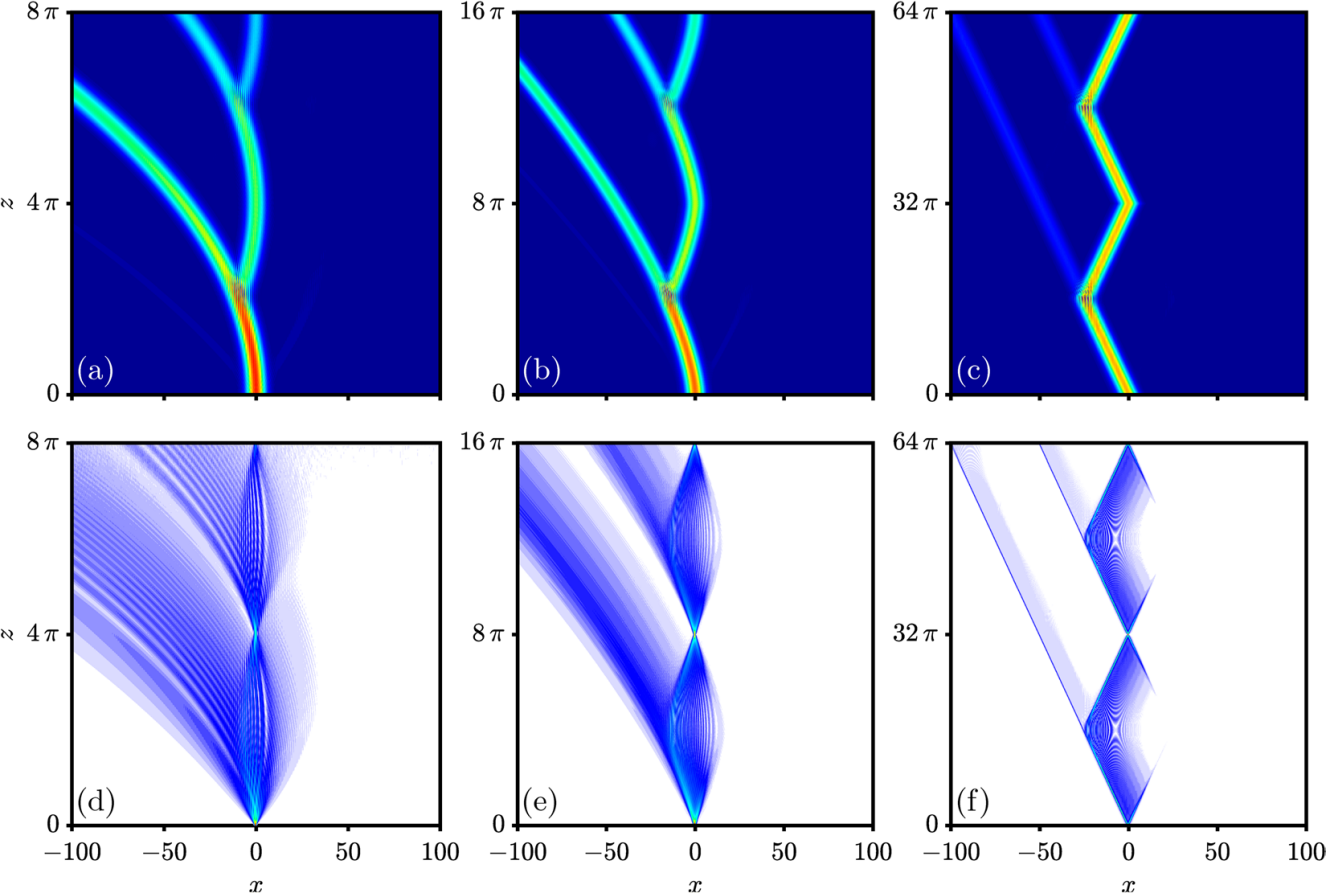
Optical Zener tunneling. (**a**)–(**c**) Wide input beam. (**d**)–(**f**) Narrow input beam. (**a**), (**d**) *a* = 0.5 and *d*_0_ = 1. (**b**), (**e**) *a* = 0.5 and *d*_0_ = 0.5. (**c**), (f) *a* = 0.5 and *d*_0_ = 0.125.

**Figure 3:**
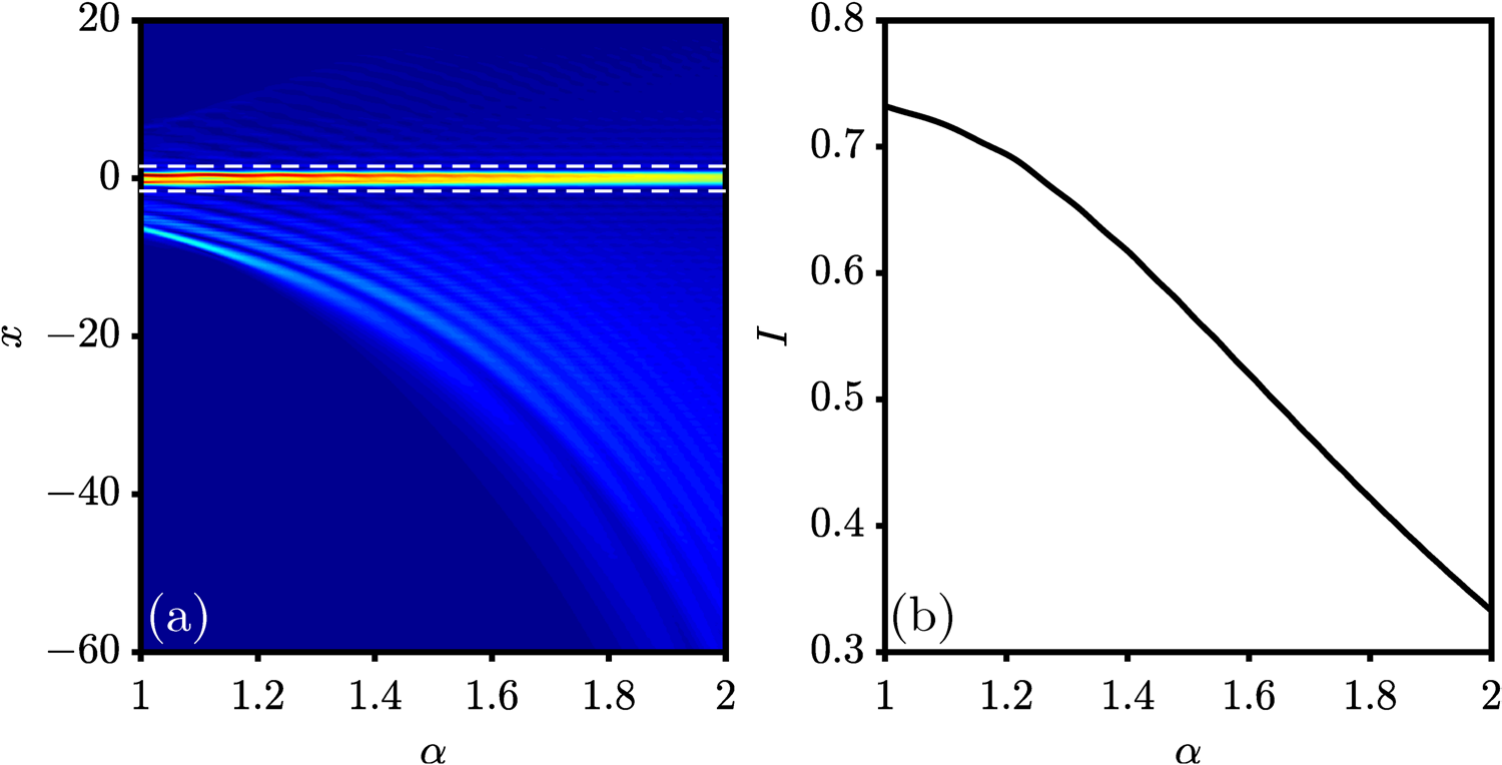
Suppression of OZT in FSE. (**a**) Output intensity versus *α*. (**b**) Intensity of the peak [indicated between two dashed lines in (**a**)] versus *α*. Parameters are the same as those used in Fig. 2(d).

**Figure 4:**
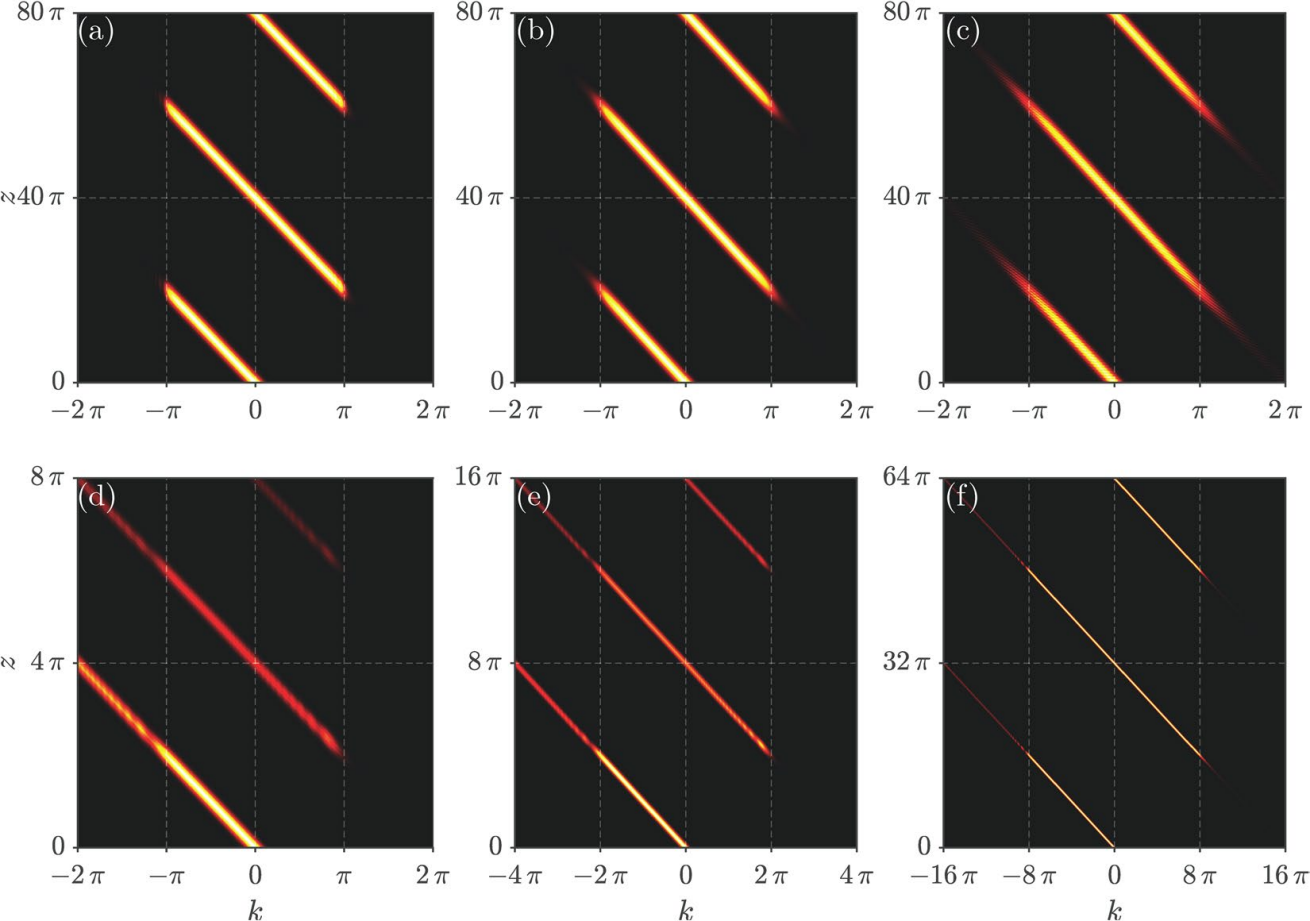
Propagation in Fourier space. (**a**)–(**c**) OBO, corresponding to Fig. 1(a)–(c). (d)–(f) OZT, corresponding to Fig. 2(a)–(c).

Our results are presented in Figs 1–4. The results concerning OBO are shown in Fig. 1, in which the strength of the linear potential equals *a* = 0.05 and the modulation of the periodic potential equals *d*_0_ = 1 (for notation, see the Methods section). With a small influence of the transverse force, coming from a weak linear potential, the band structure of the periodic potential can be calculated using the plane-wave expansion method. We first set the Lévy index to *α* = 2, corresponding to a regular Laplacian, and then calculate the (well-known) band structure, displayed in Fig. 1(a). For the fractional cases, we adopt two values of *α*: an intermediate value of *α* = 1.5 and the limiting value of *α* = 1; the results are shown in Fig. 1(b) and (c). From Fig. 1(a) to (c), one may see that the first band changes gradually from parabolic to linear (especially around *k* = 0) and that the vertical width of the band decreases (4.4539 → 2.3267 → 1.1563). Since the phenomenon of OBO is due to the Bragg refection in one band, the shape of the band will influence the OBO greatly. Based on the split-step Fourier transform method (see the Methods section), the OBOs formed during propagation are displayed in Fig. 1(d)–(f), from which one finds that the beam is compressed from $\sim 100$, to $\sim 50$ and $\sim 25$ units in the transverse direction, respectively. Another interesting feature is that the trajectory of the beam during propagation indicates the profile of the band, which is elucidated by using the dashed curves in each panel. The two features agree well with the aforementioned formation mechanism – OBO indeed reflects the properties of the band: (i) the band width is halved and so is the spreading area of the beam, and (ii) the trajectory of the beam during propagation follows the shape of the band (an intraband behavior). Considering that the properties of the band can be controlled by adjusting the Lévy index, one may claim that the Lévy index can be used to manipulate the OBO effectively. In other words, an effective manipulation of the OBO can be achieved by utilizing FSE.

### Optical Zener tunneling

If the transverse force coming from the linear potential is large enough, the tunneling between different bands will occur, and the interband phenomenon of the Zener tunneling will take place. To observe optical Zener tunneling (OZT) in FSE, we increase the value of *a* from 0.05 to 0.5; the results are shown in Fig. 2.

Similar to what was done for OBO, we start from the regular case of *α* = 2, as displayed in Fig. 2(a). There, one observes a strong OZT at the Bragg refection points, which dampens the OBO during propagation. For the fractional case of *α* = 1.5, as shown in Fig. 2(b), a significant OZT still occurs, together with a damped OBO, and the trajectory of the OBO again indicates the profile of the band, i.e., it is not parabolic as in Fig. 2(a). By further decreasing the Lévy index to the limiting case of *α* = 1, one can still observe OZT, but much suppressed [Fig. 2(c)]. The trajectory of the OBO becomes a zigzag line, due to the band becoming almost symmetric and linear in the first Brillouin zone, according to the parameters used in Fig. 2(c). Similar results have been reported earlier^33,36^. Since the trajectory of the beam is almost linear at the Bragg reflection point, the leaked OZT is along a linear trajectory, which is different from the cases seen in Fig. 2(a) and (b).

We would like to point out that many channels become excited by a wide Gaussian beam in Fig. 2(a)–(c). To contrast, we also investigate the case when only one channel is excited; the results are shown in Fig. 2(d)–(f). We pay special attention to the limiting case of *α* = 1, depicted in Fig. 2(f). The beam spreads linearly during propagation and then is reflected to form a rhombus-like pattern. At the reflection point, a bit of energy is shedded, to form OZT. Numerical simulations demonstrate that OZT for the fractional case is practically forbidden, if one uses the same parameters as for the regular case in Fig. 2(d). In addition, the beam can be well localized in the band of the fractional case, although the band width is significantly smaller than the regular case. Thus, essentially diffractionless propagation can be achieved even though one deals with a linear optical process.

## Discussion

It is interesting to check beam propagation in the Fourier space. For OBO, the beam will be confined to the first Brillouin zone (FBZ) [−*π*/*d*_0_,*π*/*d*_0_], while for OZT, the beam will escape to the higher Brillouin zone. Numerical simulations are displayed in Fig. 4.

Corresponding to the OBO displayed in Fig. 1(a)–(c), we display the same results in the Fourier space in Fig. 4(a)–(c), respectively. One can clearly see that the energy of the beam is indeed confined to the FBZ during propagation. Thus, (i) the momentum of the beam increases linearly from 0 under the action of the transverse force, (ii) the momentum changes its sign and the beam jumps across the FBZ when it undergoes the Bragg reflection at *k* = *π*/*d*_0_ (see the Methods section), (iii) the momentum continues to increase linearly until it reaches 0. The processes (i)–(iii) then repeat periodically. But, the confinement in the regular case is better than in the fractional case. The potential explanation is that the band for the fractional case becomes linear gradually with decreasing the Lévy index *α*, so the slope (the “speed” of the beam, *dβ*/*dk*) around the Bragg reflection point is bigger for the fractional case, which would help some energy escape to the higher band. However, such a situation will not hold if one adopts other parameters, e.g., the bigger value of *a* (viz. a larger transverse force), which will lead to pronounced OZT during propagation. In general, it is more difficult to observe OZT in the fractional case. The potential explanation for this phenomenon is that the beam in the regular case acquires an “acceleration” (the second-order derivative of the band, *d* ^2^ *β*/*dk* ^2^, is not zero) under a transverse force. The bigger the *a*, the bigger the “acceleration”, therefore OZT happens more easily in the regular case. Such an “acceleration” in the fractional case becomes smaller with decreasing the Lévy index.

To qualitatively display the suppression of OZT in FSE, we present in Fig. 3(a) the beam intensity distribution at the output position *z* = 4*π* [one Bloch oscillation period; the parameters are same as those used in Fig. 2(d)] as a function of *α*. Clearly, one observes that with increasing *α*, more and more beam intensity leaks out, to form OZT. Therefore, the intensity of the main peak, i.e., the recovered input beam, decreases with increasing *α*. In Fig. 3(b), we exhibit the intensity of the main peak *I* = ∫_*D*_|*ψ*|^2^ *dx* where *D* indicates the region marked by the two dashed lines in Fig. 3(a). As expected, the intensity of the main peak decreases monotonously with increasing *α*. In other words, the adjustment of the Lévy index can effectively control the emergence of OZT – that is, the OZT in FSE can be effectively suppressed by *α*.

Corresponding to the OZT in Fig. 2(a)–(c), the same results in the Fourier space are shown in Fig. 4(d)–(f). One observes that the beam energy indeed leaks to the higher-order Brillouin zone when it reaches the left boundary (−*π*/*d*_0_) of the FBZ. This indicates the transfer of energy between different bands during propagation, i.e., the OZT in the real space. Note that the OZT is most pronounced in the regular Schrödinger equation, and then it is gradually reduced in the FSE. We also mention that even though the OBO and OZT when only one channel is excited will lead to a wider beams in the Fourier space, most of the energy will still be confined to the FBZ; hence, we do not show these results here. Also, one may introduce an initial momentum −*π*/*d*_0_ < *k*_i*n*_ < *π*/*d*_0_ to the input beam. In this case, the OBO and OZT phenomena will still exist, but the Bragg reflection point will shift from ${\mathscr{D}}\mathrm{/2}$ to $\mathrm{[1/2}+{d}_{0}{k}_{{\rm{in}}}\mathrm{/(2}\pi )]{\mathscr{D}}$ (see the Methods section). These effects will not be pursued in this paper.

In summary, we have investigated the OBO and OZT in FSE. The fractional Laplacian will not only decrease the spreading of OBO, but also suppress the formation of OZT. As a result, the findings obtained in this paper may help in the design of optical switches and interconnects, by allowing the manipulation of band structure with the adjustment of the Lévy index.

## Methods

### Band structure – the plane wave expansion method

The governing FSE in dimensional units for the scalar optical field is of the form:

1 $$i\frac{{\rm{\partial }}\psi (x,z)}{{\rm{\partial }}z}=\frac{1}{2}{(-\frac{{{\rm{\partial }}}^{2}\psi (x,z)}{{\rm{\partial }}{x}^{2}})}^{\alpha /2}\,+\,[V(x)+ax]\psi (x,z),$$

where *V* = cos(2*π*/*d*_0_ *x*) is the periodic potential with *d*_0_ modulating the period, and *a* determines the strength of the transverse linear potential, modeling an external dc force. In Eq. (1), *α* is the Lévy index (1 < *α* ≤ 2). When *α* = 2, one recovers the usual Schrödinger equation. Since the potential in Eq. (1) is periodic and there is a transverse potential gradient, light beams will exhibit OBO behavior during propagation, and the corresponding oscillation period is

2 $${\mathscr{D}}=\frac{2\pi }{a{d}_{0}}\mathrm{.}$$

Without considering the last term *axψ*, the solution of Eq. (1) can naturally be written in the form *ϕ*_*n*_(*x*, *k*)exp[*iβ*_*n*_(*k*)*z*], in which *ϕ*_*n*_(*x*, *k*) is the Bloch mode and *β*_*n*_(*k*) is the propagation constant. Plugging this ansatz into Eq. (1), one obtains

3 $$-\beta \varphi +[-\frac{1}{2}{(-\frac{{{\rm{\partial }}}^{2}}{{\rm{\partial }}{x}^{2}})}^{\alpha /2}-V(x)]\varphi =0.$$

According to the Floquet-Bloch theorem, *ϕ*(*x*) can be written as *ϕ*(*x*) = *w*_*k*_(*x*)exp(*ikx*), where *w*_*k*_(*x*) = *w*_*k*_(*x* + *d*_0_) is spatially periodic. One can expand *w*_*k*_(*x*) and the potential in series of plane-waves, *w*_*k*_(*x*) = ∑_*n*_ *c*_*n*_exp(*iK*_*n*_ *x*), with *K*_*n*_ = 2*πn*/*d*_0_ and *V*(*x*) = ∑_*m*_ *P*_*m*_exp(*iK*_*m*_ *x*), where ${P}_{m}={\int }_{{d}_{0}}V(x)\,\exp \,(-i{K}_{m}x)dx/{d}_{0}$. Plugging these series into Eq. (3), one obtains

4 $$\sum _{n}[\beta +\frac{1}{2}|k+{K}_{n}{|}^{\alpha }]{c}_{n}\,\exp \,[i(k+{K}_{n})x]+\sum _{m,n}{P}_{m}{c}_{n}\,\exp \,[i(k+{K}_{n}+{K}_{m})x]=0.$$

Multiplying the above equation by exp[−*i*(*k* + *K*_*q*_)*x*] and integrating over *x* ∈ (−∞, +∞), one ends up with

5 $$-\frac{1}{2}|k+{K}_{q}{|}^{\alpha }{c}_{q}-\sum _{m}{P}_{m}{c}_{q-m}=\beta {c}_{q},$$

which is an eigenvalue problem in matrix form. By solving it, one obtains the band structure. By choosing different values of *α*, one obtains the corresponding band structures of FSE.

### Propagation – the split-step Fourier transform method

The propagation is executed using the split-step Fourier transform (FT) method, which demands a separate treatment of the diffraction term and the potential term,

6 $$i\frac{\partial \psi (x,\,z)}{\partial z}=(\hat{D}+\hat{P})\psi (x,z),$$

in which

$$\hat{D}=\frac{1}{2}{(-\frac{{{\rm{\partial }}}^{2}}{{\rm{\partial }}{x}^{2}})}^{\alpha /2}\,{\rm{a}}{\rm{n}}{\rm{d}}\,\,\hat{P}=V(x)+ax.$$

Doing the FT of the diffraction term, one obtains

7 $$i\frac{\partial \tilde{\psi }(k,z)}{\partial z}=\frac{1}{2}|k{|}^{\alpha }\tilde{\psi }(k,\,z),$$

where $\tilde{\psi }$ is the FT of *ψ*. The solution of Eq. (7) after one step can be written as

8 $$\mathop{\psi }\limits^{ \sim }(k,\,z+dz)=\exp (-\frac{i}{2}|k{|}^{\alpha }dz)\mathop{\psi }\limits^{ \sim }(k,\,z).$$

By performing inverse FT, one obtains *ψ*(*x*, *z* + *dz*). Considering that the potential term gives the solution after one step

9 $$\psi (x,\,z+dz)=\exp \{-i[V(x)+ax]dz\}\,\psi (x,\,z),$$

one achieves the whole propagation step by step. This one-step FT method is the lowest-order method in *dz*. In a more accurate procedure, one must take into account that the operators $\hat{D}$ and $\hat{P}$ do not commute with each other. To improve the accuracy, higher-order split-step FT methods are introduced, which go by the name of split-step symplectic algorithms.

### Bragg reflection condition

Generally, the Bragg reflection condition is

10 $$2{d}_{0}\,\sin \,\theta =n\lambda ,$$

in which *λ* is the wavelength, and *n* = ±1, ±2, … is an integer, the order of Bragg reflection. Since the transverse force is exerted along the periodic potential, the incident angle is *θ* = *π*/2; therefore one ends up with

11 $$k=n\frac{\pi }{{d}_{0}},$$

where *k* = 2*π*/*λ*. We consider the principal reflection, so we adopt *n* = ±1.

However, if there is an initial momentum *k*_i*n*_ in the incident beam (the incident angle is still *θ* = 0), the transverse force will first overcome it over the propagation distance

12 $${z}_{{\rm{in}}}=\frac{{k}_{{\rm{in}}}{d}_{0}}{2\pi }{\mathscr{D}},$$

regarding the width of the first Brillouin zone 2*π*/*d*_0_. As a result, the Bragg reflection point moves from ${\mathscr{D}}/1$ to

$$(\frac{1}{2}+\frac{{k}_{{\rm{i}}{\rm{n}}}{d}_{0}}{2\pi }){\mathscr{D}}.$$
